# The Role of Camels in the Epizootiology and Epidemiology of Plague in the Republic of Kazakhstan

**DOI:** 10.3390/pathogens15070669

**Published:** 2026-06-25

**Authors:** Raikhan Mussagalieva, Ziyat Abdel, Zauresh Zhumadilova, Aigul Abdirassilova, Svetlana Issaeva, Bolatbek Baitursyn, Nurbol Shaki, Beck Abdeliyev, Dinmukhammed Otebay, Tatyana Meka-Mechenko

**Affiliations:** M. Aikimbayev’s National Scientific Center for Especially Dangerous Infections, 14 Zhakhanger St., Almaty A35P0K3, Kazakhstan; raikhansafar@gmail.com (R.M.); abdelziyat767@gmail.com (Z.A.); zzbgdirect@nscedi.kz (Z.Z.); aigul.abdirassilova@mail.ru (A.A.); s.isaeva64@mail.ru (S.I.); b.bola-1993@mail.ru (B.B.); nurbol.shakiy@gmail.com (N.S.); abdelbeck@gmail.com (B.A.); dimash12.95@mail.ru (D.O.)

**Keywords:** plague, *Yersinia pestis*, camels, epizootology, epidemiology, Kazakhstan, natural foci, GIS, risk analysis, One Health

## Abstract

Camels are increasingly recognized as an important epizootological and epidemiological link in natural plague foci, contributing to the transmission of *Yersinia pestis* from wildlife to humans. In the Republic of Kazakhstan, where natural plague foci occupy up to 40% of the territory, the rapid growth of camel populations may significantly enhance epidemiological risks. The aim of this study was to perform a comprehensive assessment of the role of camels in the epizootology and epidemiology of plague based on retrospective data (1907–2003) and contemporary monitoring (2000–2025), including spatial analysis and risk zoning. A total of 64 cases of camel plague and 43 epizootic foci were identified during the historical period. More than 400 human cases, including fatal outcomes, associated with infected camels were documented, with direct contact during slaughter and meat processing accounting for 94.7% of infections. Spatial analysis and epidemiological zoning revealed a heterogeneous risk distribution, with western and southern regions representing the highest-risk areas. Serological investigation (*n* = 2726) showed 75.6% seropositivity, likely reflecting substantial population immunity largely associated with vaccination. Despite increasing camel population size (from 227.7 to 304.0 thousand heads in 2020–2025), vaccination coverage varied between 32.0% and 51.0%, reflecting risk-based preventive strategies. The absence of recent camel plague cases supports the effectiveness of integrated control measures, including vaccination, surveillance, and the establishment of protective zones. These findings suggest that camels remain an important component of plague transmission systems and should be systematically integrated into surveillance programs within a One Health framework.

## 1. Introduction

Plague remains one of the most significant especially dangerous infectious diseases, characterized by persistent natural foci and posing a continuous threat to public health in many regions of the world. Despite advances in medicine and epidemiological surveillance systems, human cases continue to be reported annually, predominantly in countries in Africa and Asia [1,2].

The causative agent of plague, *Y. pestis*, circulates within complex natural focal systems involving wild rodents and their ectoparasites (fleas), ensuring the long-term persistence of the infection in natural ecosystems [3,4]. Transmission to humans occurs primarily via vector-borne routes; however, in certain situations, secondary hosts, including domestic animals, play an important role as intermediate links between natural foci and human populations [5].

Among such animals, camels are of particular importance due to their susceptibility to *Y. pestis* and their potential to become infected through contact with plague-endemic environments. Infection in camels may occur in acute or septicemic forms and is often associated with high mortality, making them a potentially significant source of human infection [6,7]. Numerous cases of human plague transmission associated with the slaughter and processing of infected camels have been reported in the literature, including cases involving severe clinical forms of the disease [8,9].

This issue is particularly relevant in Central Asian countries, including the Republic of Kazakhstan, where natural plague foci occupy a substantial portion of the territory. The total area of enzootic regions is estimated to reach approximately 1.1 million km^2^, accounting for up to 40% of the country’s territory [10].

These natural plague foci are predominantly associated with desert, semi-desert, and steppe ecosystems characterized by persistent circulation of *Y. pestis* among wild rodent reservoirs and flea vectors [11]. In Kazakhstan, camel husbandry is traditionally concentrated in western and southern regions, which coincide with areas of active natural plague foci. In recent decades, a significant increase in camel population size has been observed, driven by the development of the agricultural sector and the growing economic importance of camel breeding [12]. According to official statistics, the camel population has more than tripled over the past 20–25 years, reaching approximately 300,000 animals by 2025 [13].

Historical data indicate a substantial epidemiological role of camels in Kazakhstan. Between 1907 and 2003, dozens of cases of plague in camels were recorded, accompanied by numerous human infections, primarily associated with contact with infected animals [14,15,16]. Analysis of these cases suggests that camels can serve as an important epizootological and epidemiological link, facilitating the transmission of the pathogen from natural foci to human populations [17].

Despite the accumulated evidence, the role of camels under contemporary conditions remains insufficiently studied, particularly in the context of changes in livestock management systems, climatic factors, and administrative-territorial restructuring. Moreover, there is a lack of comprehensive studies integrating retrospective analysis, current epizootological data, and spatial risk zoning.

In this regard, the aim of the present study is to provide a comprehensive assessment of the role of camels in the epizootology and epidemiology of plague in the Republic of Kazakhstan using retrospective data, contemporary statistical materials, and spatial analysis approaches, as well as to differentiate territories according to epidemiological risk levels.

## 2. Materials and Methods

### 2.1. Study Design and Data Sources

The study was based on an integrated analysis of retrospective (1907–2003) and contemporary (2000–2025) data on camel populations, plague epizootics, and epidemiological surveillance in Kazakhstan.

Data sources included archival epidemiological and epizootological records, official statistical data on camel population dynamics, veterinary service reports on vaccination and control measures, as well as laboratory data obtained from serological investigations.

### 2.2. Serological Analysis

In 2024–2025, a total of 2726 camel serum samples were collected from 2726 animals located in 115 rural districts across eight regions of Kazakhstan (West Kazakhstan, Atyrau, Mangystau, Aktobe, Kyzylorda, Turkestan, Zhambyl, and Almaty regions) as part of routine epizootological surveillance activities. Of these, 1463 samples from 61 rural districts were collected in 2024, and 1263 samples from 54 rural districts were collected in 2025. The sampled animals were maintained in different epizootological units situated within plague-enzootic territories characterized by moderate to high epizootic activity.

Biological samples were collected from camel farms located in epidemiologically relevant areas where camel breeding overlaps with natural plague foci. Within each herd, animals were selected using a convenience-based field sampling approach aimed at obtaining representative coverage of different age groups and management conditions.

Clinically healthy animals older than 6 months of age were included in the study. Very young animals (<6 months) were excluded to minimize the influence of maternally derived antibodies. Animals with severe clinical illness unrelated to plague at the time of sampling were not included. Both male and female camels were sampled. Pregnant females were not specifically excluded unless handling was considered unsafe by veterinary personnel.

All serum samples were labeled and processed according to standardized veterinary laboratory protocols. Serological testing was performed using the indirect hemagglutination assay (IHA) with F1 antigen for the detection of antibodies to *Y. pestis*. The assay was performed using a plague erythrocyte antigen diagnostic kit (National Scientific Center for Especially Dangerous Infections named after M. Aikimbayev, Almaty, Kazakhstan). The test is based on formalin-fixed sheep erythrocytes sensitized with the capsular F1 antigen of *Y. pestis*. Serum samples were heat-inactivated, adsorbed with formalinized sheep erythrocytes to reduce non-specific reactions, and tested by serial two-fold dilutions. Sensitized erythrocytes were added to each well and incubated at room temperature for 2–3 h. Positive reactions were identified by diffuse hemagglutination (“umbrella” pattern), whereas negative reactions produced a compact erythrocyte button. Samples with antibody titers ≥1:20 were considered positive.

In parallel, molecular genetic testing using real-time PCR was performed to detect *Y. pestis* DNA.

Samples with doubtful or borderline agglutination reactions were re-tested using the same assay protocol. Only samples demonstrating reproducible positive reactions at diagnostic titers were included in the final analysis.

Laboratory records and serological results were independently cross-checked by two investigators prior to statistical analysis to minimize transcription and reporting errors.

The majority of sampled herds were located in western and southern Kazakhstan, reflecting the regional concentration of camel populations and the distribution of plague-endemic territories. Consequently, regions with low camel density were proportionally less represented in the survey, consistent with the risk-based design of epizootological surveillance activities.

The individual apparent seroprevalence was calculated as the proportion of seropositive camels among all tested animals. Exact 95% confidence intervals (95% CIs) were calculated using the binomial distribution. Herd-level prevalence was not estimated because information on herd structure and ownership was unavailable in the national surveillance database used in this study. Therefore, the analysis was limited to individual animal seroprevalence.

### 2.3. Spatial and Statistical Analysis

Spatial analysis and epidemiological zoning were conducted using GIS-based approaches to assess the geographic distribution of camel populations, plague-endemic territories, historical epizootic activity, and human plague cases in Kazakhstan. Regions were classified according to camel population size and density, the degree of overlap with natural plague foci, historical epizootic activity, and occurrence of human cases.

Risk levels were categorized into four classes: very high-, high-, moderate-, and low-risk.

All spatial analyses and cartographic visualizations were performed using ArcGIS 10.8 (Esri, Redlands, CA, USA).

### 2.4. Epizootological Monitoring and Control Measures

Data on vaccination, epizootic detection, and protective zones (2020–2025) were analyzed.

Vaccination coverage (%) was calculated as:

(1) $$Coverage=\frac{Vaccinated\;animals}{Total\;population}\times 100\%$$

## 3. Results

### 3.1. Dynamics of Camel Population in Kazakhstan (2000–2025)

**Figure 1:**
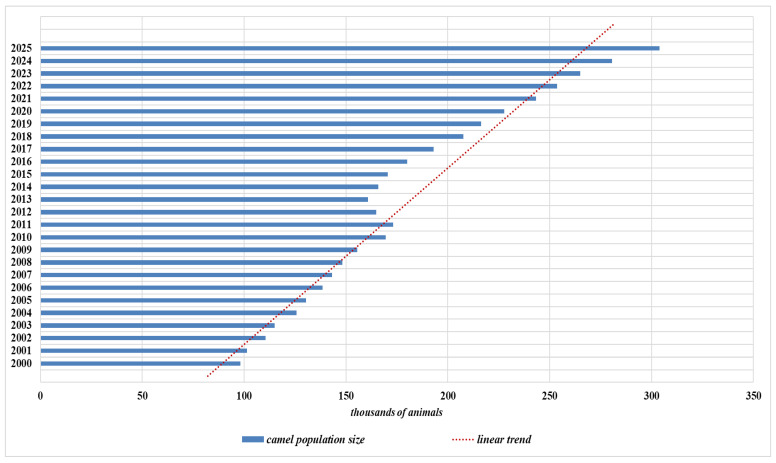
Temporal dynamics of camel population growth in Kazakhstan during 2000–2025 with linear trend analysis.

Analysis of camel population dynamics in Kazakhstan over the period 2000–2025 revealed a sustained upward trend in herd size (Figure 1).

At the beginning of the study period (2000), the camel population was approximately 98,000 animals. This was followed by a gradual increase, with minor fluctuations observed in individual years. From 2010 onward, the growth rate became more pronounced, and since 2015, a clearly defined positive trajectory has been observed, reflecting the active development of the camel husbandry sector.

By 2020, the population had reached approximately 216,000 animals, and by 2025, it had increased to about 300,000–304,000, representing more than a threefold increase compared to 2000. Trend analysis indicates that during the initial period (2000–2010), population growth followed a relatively moderate trajectory, whereas in subsequent years (2015–2025) growth became more pronounced.

The increase in the camel population in Kazakhstan is driven by a combination of ecological, economic, and organizational factors. Camels exhibit a high level of adaptation to extreme environmental conditions typical of desert and semi-desert regions, efficiently utilizing limited forage resources. This makes them one of the most resilient livestock species in arid ecosystems. The primary regions of camel breeding—western and southern Kazakhstan—are characterized by extensive grazing areas, a significant proportion of which overlap with natural plague foci.

An additional driver of population growth is the increasing economic importance of camel husbandry, associated with rising demand for camel products (milk, meat, and wool), as well as state-supported agricultural development programs. At the same time, specific management practices, including predominantly free-ranging grazing systems and prolonged exposure of animals to natural environments, increase the likelihood of contact with infectious agents.

It should also be noted that in remote regions, elements of traditional livestock management persist, including the slaughter of animals in field conditions without prior veterinary inspection. Such practices further increase epidemiological risks.

Thus, the sustained increase in camel population, particularly in regions overlapping with natural plague foci, leads to a higher probability of contact between animals and *Y. pestis*, thereby enhancing the potential role of camels in both epizootic and epidemiological processes.

### 3.2. Historical Epidemiological Role of Camels in Plague Transmission (1907–2003)

A retrospective analysis of archival epidemiological and epizootological data demonstrated that, during the period 1907–2003, camels played a significant role in the epidemiology of plague in Kazakhstan.

During this period, a total of 64 cases of plague in camels were recorded, along with 43 identified epizootic foci. In addition, more than 400 human cases and deaths associated with infected camels were documented. Analysis of transmission pathways indicated that 94.7% of human infections were associated with direct contact during slaughtering, carcass processing, and meat handling of infected camels.

Spatial analysis revealed that the highest concentration of epidemiologically significant cases occurred in the western and southern regions of the country, particularly in the Mangystau, Atyrau, and Kyzylorda regions. These areas correspond to zones of active natural plague foci and high population densities of primary reservoir hosts.

The regional distribution of camel plague cases and associated epizootic foci is summarized in Table 1.

Analysis of transmission pathways indicated that the overwhelming majority of human infections (94.7%) were associated with direct contact with infected camels, primarily during slaughter, carcass processing, and the consumption of meat without adequate thermal treatment. This transmission route reflects the specific features of traditional livestock management and the handling of animal products in endemic regions.

Clinical manifestations of plague in camels were predominantly nonspecific and included fever, depression, reduced activity, and enlargement of lymph nodes. Such a nonspecific presentation significantly complicated timely diagnosis and contributed to the inclusion of infected animals in routine livestock production and consumption systems.

Overall, historical data clearly demonstrate that camels serve as an important epidemiological link in the transmission of *Y. pestis* to humans, primarily via contact and alimentary routes, particularly in the absence of timely veterinary control and diagnostic measures.

### 3.3. Spatial Zoning of Camel Population and Plague-Endemic Areas

**Figure 2:**
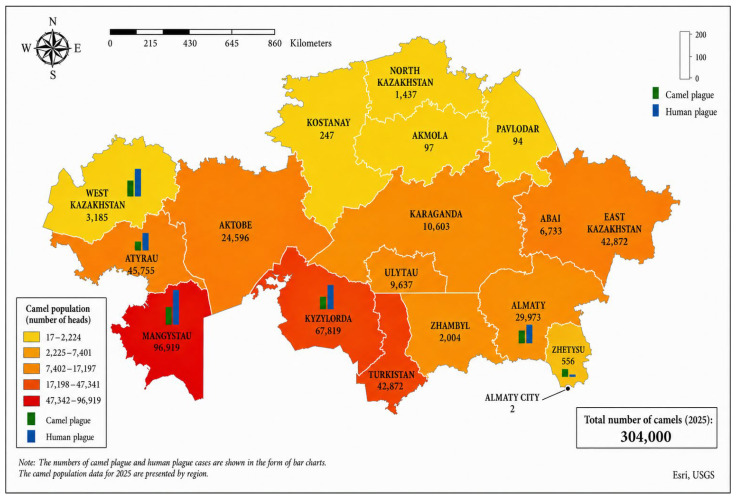
Epidemiological zoning of Kazakhstan based on camel population density (2025), integrated with plague-endemic areas and historical outbreak patterns.

Spatial analysis of camel population distribution in relation to natural plague foci enabled the differentiation of the territory of Kazakhstan according to epizootological characteristics (Figure 2).

The results indicate that camel distribution is markedly heterogeneous and closely associated with arid ecosystems, where the principal natural plague foci are located. The highest concentration of camels is observed in the western and southern regions of the country, including the Mangystau, Atyrau, Kyzylorda, and Turkestan regions.

The summarized results of spatial zoning, including the quantitative and geographic characteristics of the identified zones, are presented in Table 2.

The implemented zoning approach has fundamental importance for optimizing epizootological and epidemiological surveillance systems. Differentiation of territories according to the intensity of the epizootic situation allows for a more targeted allocation of monitoring resources in high-risk regions and improves the effectiveness of veterinary and sanitary preventive measures, including vaccination and the control of animal movement. In addition, spatial zoning facilitates the early identification of areas with potential activation of natural foci and contributes to reducing the risk of *Y. pestis* transmission from animals to humans.

This approach is particularly important under conditions of extensive camel husbandry and free-ranging grazing systems, where direct contact between animals and natural reservoirs of infection is unavoidable and creates persistent conditions for maintaining the epizootic process.

Thus, epidemiological zoning represents an effective tool for risk-based management of natural focal infections and provides a necessary framework for planning surveillance and preventive measures in enzootic territories. The spatial patterns identified in this study confirm the formation of stable high-risk areas in which camels constitute an important component of the plague epizootic system.

### 3.4. Epidemiological Risk Assessment of Camel-Associated Plague

An integrated analysis combining spatial distribution patterns of camel populations (Section 3.1), the results of epidemiological zoning (Table 2), and historical epizootological data (Section 3.2) enabled a comprehensive assessment of the epidemiological risk associated with the involvement of camels in the circulation of *Y. pestis* across Kazakhstan.

The results indicate that the formation of risk zones is strongly spatially structured and determined by the combined influence of several factors, including camel population size and density, the degree of overlap with natural plague foci, the presence of historically recorded epizootics, and livestock management practices.

**Figure 3:**
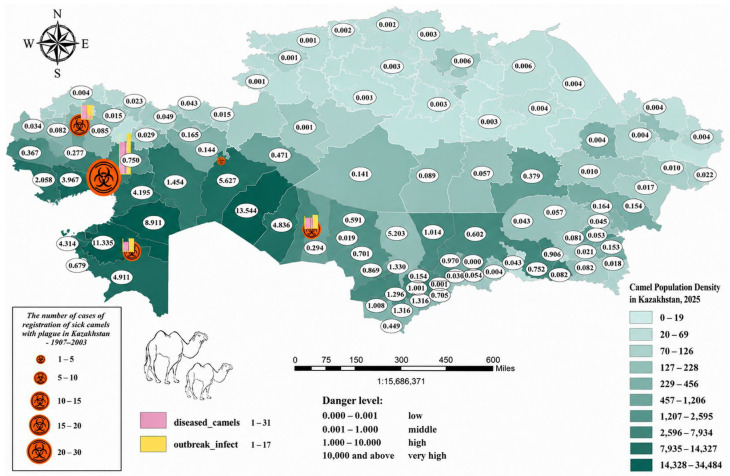
Epidemiological risk zoning of Kazakhstan based on camel population density (2025), integrated with plague-endemic areas and historical camel plague outbreak data (1907–2003).

According to the classification presented in Figure 3, the territory of Kazakhstan was differentiated into four levels of epidemiological risk.

The criteria for classification and the characteristics of each risk category reflect the spatial heterogeneity of the epizootic situation.

Very high-risk areas. These include specific parts of the western regions, primarily the Mangystau and Atyrau regions, where the most unfavorable factors converge. These areas are characterized by extremely high camel density, extensive enzootic territories, and a high concentration of historical epizootics, including the presence of epidemiological “hotspots.”

High-risk areas. This category includes the Kyzylorda region and parts of the Turkestan region, where camel populations remain high and significantly overlap with natural plague foci. These areas are characterized by high livestock density, active natural foci, and previously documented human infections.

Moderate-risk areas. These include the Aktobe, Karaganda, Almaty, Zhetysu, Zhambyl, Ulytau, and Abai regions. They are characterized by moderate camel population density, partial overlap with enzootic territories, and the potential for localized epizootic events.

Low-risk areas. These include northern regions and major urban centers (Akmola, Pavlodar, Kostanay, and North Kazakhstan regions, as well as Astana, Almaty, and Shymkent). These areas are characterized by low camel density, minimal or absent natural plague foci, and a lack of historically significant cases.

The spatial distribution of risk levels (Figure 3) demonstrates that the most unfavorable epidemiological conditions are concentrated in regions with high camel density and maximal overlap with natural plague foci in Kazakhstan.

Thus, the risk of camel involvement in the epizootic process and the subsequent transmission of *Y. pestis* to humans exhibits a clearly defined spatial structure, reaching its highest levels in the western regions of Kazakhstan, where biological, ecological, and livestock-management factors interact.

### 3.5. Serological Status and Vaccination Coverage in the Camel Population

To assess serological evidence of *Y. pestis* exposure and population immunity in camels, serological investigations using the indirect hemagglutination assay (IHA) with F1 antigen were conducted in 2024–2025. In parallel, molecular genetic testing using real-time PCR was performed to detect the presence of *Y. pestis* DNA in sampled animals.

A total of 2726 serum samples were collected from 2726 camels located in 115 rural districts across eight regions of the Republic of Kazakhstan (West Kazakhstan, Atyrau, Mangystau, Aktobe, Kyzylorda, Turkestan, Zhambyl, and Almaty regions). Of these, 1463 samples from 61 rural districts were collected in 2024, and 1263 samples from 54 rural districts were collected in 2025.

Overall, 2060 samples were positive for antibodies against the F1 antigen of *Y. pestis*, corresponding to an individual apparent seroprevalence of 75.6% (95% CI: 73.9–77.2%).

The sampled animals originated from different epizootological units situated within plague-enzootic territories characterized by moderate to high epizootic activity.

Biological samples were collected from camel farms located in epidemiologically relevant areas where camel breeding overlaps with natural plague foci. Among seropositive animals, antibody titers ranged from 1:20 to 1:320. Regional differences in seropositivity and antibody titer distribution are summarized in Table 3.

The distribution of titers indicates heterogeneity in the immune response. Low titers (1:20–1:40) likely reflect residual or post-vaccination immunity, whereas medium to high titers (≥1:80) may reflect varying levels of immune response, including possible recent antigenic stimulation.

All real-time PCR results for the detection of *Y. pestis* DNA were negative. No molecular evidence of active *Y. pestis* infection was identified among the sampled animals.

These serological findings should be interpreted with caution, as the field sampling design was risk-based rather than strictly probabilistic.

The dynamics of camel population size, vaccination coverage, and epizootological control measures during 2020–2025 are presented in Table 4.

Analysis shows that, against the background of a steady increase in the camel population (from 227.7 to 304.0 thousand animals), a gradual decline in relative vaccination coverage was observed between 2020 and 2024 (from 38.6% to 32.0%). This trend reflects favorable epizootic conditions in preceding years and the application of a risk-based approach to preventive planning. In 2025, a substantial increase in vaccination (up to 155,059 animals) resulted in an increase in coverage to approximately 51%, indicating intensified preventive efforts in high-risk regions.

The epizootological control system in Kazakhstan includes regular detection of plague epizootics and the establishment of sanitary protection zones. Despite the relatively limited area of detected epizootics (3.0–8.9 thousand km^2^), a considerable number of protective zones (116–196 annually) are established each year to localize infection foci. Within these zones, comprehensive veterinary and sanitary measures are implemented, including the treatment of territories and animals against ectoparasites, which serve as vectors of plague.

The establishment of sanitary protection zones reduces the likelihood of camel infection by fleas and other vectors of *Y. pestis*, thereby interrupting transmission pathways and decreasing the probability of animal involvement in the epizootic process. An additional component of the system is the legally mandated reporting of unexplained animal mortality. Such cases must be reported by veterinary services to anti-plague institutions for laboratory diagnosis, in accordance with national legislation, including the Law on Biological Safety of the Republic of Kazakhstan.

The absence of reported cases of camel morbidity and mortality due to plague in recent years indicates the effectiveness of this integrated prevention system, which combines vaccination, monitoring, and rapid response to detected epizootics.

Overall, the observed seropositivity likely reflects substantial post-vaccination population immunity, although natural antigenic stimulation in active foci cannot be excluded. Despite these positive outcomes, maintaining a stable level of herd immunity requires further optimization of vaccination coverage in the context of the continuing growth of the camel population. The combination of risk-based vaccination, systematic epizootological monitoring, and the establishment of sanitary protection zones contributes to the effective containment of plague activity among camels and helps prevent transmission to humans.

### 3.6. Economic Implications of Camel-Associated Plague Outbreaks

Plague outbreaks associated with livestock, particularly camels, represent not only a significant epidemiological threat but also a substantial economic burden on public health systems. The economic impact of such outbreaks extends beyond direct medical costs and includes a wide spectrum of anti-epidemic interventions, logistical operations, and long-term surveillance activities.

According to Aikimbayev, the total cost of an epidemic response to plague depends on the clinical form of the disease, the number of exposed individuals, and the scale of containment measures required. In severe scenarios, particularly in cases of suspected pneumonic plague, large-scale interventions involving multiple response units may be necessary, significantly increasing overall expenditures. For example, outbreak response activities in Kazakhstan have historically required substantial financial resources, reaching levels equivalent to hundreds of thousands of USD, depending on the scale and urgency of intervention [18].

A detailed economic assessment of plague response conducted in the Kyzylorda region (2003) provides a quantitative breakdown of expenditures associated with a single detected case. The total cost of anti-epidemic measures amounted to 4,541,422 tenge (~30,482 USD), demonstrating the high financial burden even for localized outbreaks [19]. The cost structure revealed that the largest expenditures were associated with the deployment of epidemiological and laboratory response teams, disinfection and decontamination measures, transportation and logistical support, household surveillance (door-to-door visits), vaccination campaigns, and administrative and operational coordination.

Notably, vaccination of at-risk populations also constituted a measurable component of the total cost. In the analyzed outbreak, the average cost of specific vaccination was estimated at 108.4 tenge per person, with more than 2000 individuals immunized [19].

In addition to direct response costs, indirect economic losses must be considered. These include productivity losses due to quarantine measures, restrictions on livestock movement, and potential impacts on rural economies that depend heavily on camel breeding and trade. Given that camels serve both as a source of infection and an economic asset in endemic regions, outbreaks can have a dual impact—affecting both public health and agricultural sustainability.

Importantly, the scale of the economic burden is strongly influenced by the timeliness of diagnosis. Delayed identification of plague cases may lead to the escalation of response measures, including the expansion of contact tracing, increased laboratory workload, and broader geographic containment zones. As highlighted in previous studies, misclassification or delayed diagnosis can result in a dramatic increase in operational costs due to unnecessary large-scale interventions [18].

From a risk management perspective, these findings emphasize the importance of early detection systems, targeted surveillance in camel-associated transmission zones, and cost-effective preventive strategies. Investments in routine epizootological monitoring, rapid diagnostic tools, and vaccination of high-risk populations may significantly reduce the overall economic burden of plague outbreaks.

Overall, the economic analysis demonstrates that even a single case of plague can trigger a cascade of high-cost interventions. In regions where camel husbandry is widespread, integrating veterinary surveillance with public health systems is essential to minimize both epidemiological and economic risks.

## 4. Discussion

The results of this study demonstrate that camels play a significant and multifaceted role in the epizootology and epidemiology of plague in Kazakhstan. Historically, camels have acted as a critical bridge between natural plague foci and human populations, particularly when infected animals were slaughtered or processed without veterinary control.

In our study, more than 94% of human infections associated with camels were linked to direct contact during slaughter, carcass processing, or the consumption of meat from infected animals. This pattern is consistent with international observations. In Libya in 1976, plague cases occurred after a sick camel was slaughtered and its meat was distributed for consumption; severe illness developed among villagers involved in handling or eating the meat [20,21]. Similar transmission was reported in Saudi Arabia in 1994, where human plague cases were associated with meat from a sick camel [22,23]. In Jordan in 1997, an outbreak of pharyngeal plague occurred after the consumption of camel meat, and 11 of 12 patients reported eating meat from the same infected camel [24]. These observations support the conclusion that camel-associated plague is not limited to Kazakhstan but represents a broader epidemiological pattern in arid and semi-arid regions.

The spatial analysis in Kazakhstan highlights a strong association between camel distribution and plague-endemic territories. More than 90% of the camel population is concentrated in regions with natural plague foci, particularly in western and southern Kazakhstan. This spatial overlap creates persistent conditions for interaction among camels, rodent reservoirs, flea vectors, and humans. Farming systems in western and southern Kazakhstan are predominantly characterized by extensive free-ranging grazing practices, prolonged seasonal animal movements, and frequent contact of camels with natural desert and semi-desert ecosystems. These conditions increase the likelihood of interaction between camels, rodent reservoirs, and flea vectors of *Y. pestis*. In addition, traditional slaughtering and carcass-processing practices performed outside centralized veterinary control may further increase the zoonotic risk associated with infected animals, particularly in remote rural areas. The WHO manual emphasizes that plague surveillance should include ecological, animal, vector, and human components, especially in endemic regions where pathogen circulation persists in natural ecosystems [25].

Our findings also correspond with earlier experimental and field observations from Central Asia. Fedorov showed that camels could become infected with plague under experimental conditions, although their susceptibility varied, indicating that camels may act as accidental but epidemiologically important hosts rather than primary reservoirs [26]. This supports the interpretation that camels are not the core reservoir of *Y. pestis*, but they can become a dangerous secondary host when grazing in active natural foci.

Despite these risk factors, no recent cases of camel plague have been recorded in Kazakhstan. This is likely explained by the effectiveness of integrated control measures, including vaccination, continuous epizootological monitoring, rapid response to detected epizootics, and the establishment of sanitary protection zones. Importantly, the observed decline in vaccination coverage between 2020 and 2024 should not be interpreted as a weakening of the system. Rather, it reflects a risk-based preventive strategy adapted to favorable epizootic conditions. The subsequent increase in vaccination in 2025 demonstrates the flexibility of the national surveillance system and its capacity to intensify measures when needed.

The establishment of sanitary protection zones plays a key role in interrupting transmission by reducing contact between camels and infected ectoparasites. These measures, together with mandatory reporting and laboratory investigation of unexplained animal deaths, constitute a comprehensive surveillance framework supported by national biosafety legislation. This approach is consistent with modern plague-control principles, where prevention relies on early detection, vector control, vaccination when indicated, and coordinated action between veterinary and public health services [25].

The observed seropositivity in sampled camel populations (75.6%) likely reflects substantial population immunity, most probably associated with vaccination, although natural antigenic stimulation in endemic areas cannot be excluded. This finding should be interpreted cautiously: seropositivity indicates an immune response, but it does not necessarily mean active infection. Therefore, serological monitoring is most valuable when interpreted together with epizootological, clinical, and spatial data.

At the same time, caution is required when interpreting the apparent decline in reported camel and human plague cases exclusively as a consequence of improved control measures. Natural fluctuations in plague activity, changes in rodent reservoir and flea vector populations, environmental and climatic variability, and long-term ecological dynamics may also influence the intensity of epizootic and epidemiological processes. Therefore, the observed reduction in reported cases likely reflects a combination of surveillance, vaccination, environmental, and ecological factors.

The economic and epidemiological assessment of plague control in Kazakhstan is consistent with the framework developed by Aikimbayev and colleagues, emphasizing the importance of integrated surveillance and comprehensive anti-epidemic measures [18,19,27].

Overall, the findings support the concept that camel-associated plague risk is governed by a complex interaction of ecological, biological, veterinary, and socio-economic factors. The Kazakhstani model differs from the Middle Eastern outbreaks, where the consumption of infected camel meat played a dominant role, but the underlying mechanism is similar: infected camels can transfer *Y. pestis* from natural ecosystems into human settings. Therefore, camel surveillance should remain an integral part of plague monitoring in Kazakhstan, particularly within a One Health framework that links human health, animal health, vector ecology, and environmental risk.

## 5. Conclusions

The present study provides a comprehensive assessment of the role of camels in the epizootology and epidemiology of plague in Kazakhstan. The results suggest that camels constitute an important epidemiological link contributing to the transmission of *Y. pestis* from natural reservoirs to humans, particularly in regions where high animal density coincides with plague-endemic territories.

Significant differences in seroprevalence were observed among regions, suggesting that exposure to *Y. pestis* may vary according to host-related and geographic factors. These results indicate a heterogeneous distribution of exposure across the sampled camel populations.

The absence of recent cases of camel plague reflects the effectiveness of integrated control measures, including systematic vaccination, continuous epizootological surveillance, and the establishment of sanitary protection zones. At the same time, the ongoing increase in camel population size, combined with extensive grazing practices and close interaction with natural ecosystems, sustains the potential for pathogen circulation and requires continued vigilance.

Importantly, the results indicate that prevention-oriented strategies are not only epidemiologically effective but also economically justified. The implementation of routine vaccination, targeted surveillance, and early response mechanisms reduces the likelihood of large-scale outbreaks and minimizes the need for resource-intensive emergency interventions.

Overall, the findings highlight the necessity of maintaining a risk-based surveillance system, adaptive vaccination strategies, and the integration of camel monitoring into national plague control programs. Strengthening these measures within a One Health framework remains essential for ensuring both epidemiological stability and the long-term sustainability of plague control in Kazakhstan.

## Figures and Tables

**Table 1:** Regional distribution of camel plague cases and associated epizootic foci in Kazakhstan (1907–2003).

Region	Camel Plague Cases (*n*)	Epizootic Foci Associated with Slaughter (*n*)	Share of Total Cases (%)	Epidemiological Interpretation
Atyrau	31	17	48.4	Major hotspot, high transmission intensity
Mangystau	9	5	14.1	High-risk desert focus
West Kazakhstan	15	13	23.4	Stable epizootic activity
Kyzylorda	8	7	12.5	Active southern focus
Aktobe	1	1	1.6	Sporadic cases
Total	64	43	100	—

**Table 2:** Zoning of Kazakhstan according to the intensity of the epizootic situation of camel plague.

Zone	Risk Level	Camel Population (Heads)	Total Area (Thousand km^2^)	Plague-Endemic Area (Thousand km^2^)	Camel Plague Cases (*n*)	Regions
I	High	20,000–100,000	953.15	732.3	51	Atyrau, Mangystau, Aktobe, Kyzylorda, Turkistan
II	Moderate	1000–20,000	1230.72	427.4	13	Almaty, Zhetysu, Zhambyl, West Kazakhstan, Karaganda, Ulytau, Abai, East Kazakhstan
III	Low	0–1000	541.03	0.0	0	Akmola, Pavlodar, Kostanay, North Kazakhstan, Almaty city, Astana, Shymkent

**Table 3:** Regional distribution of serological and molecular surveillance results in camels from plague-endemic territories of Kazakhstan (2024–2025).

Region	Samples Collected (*n*)	Rural Districts Surveyed (*n*)	Seropositive Samples (%)	Antibody Titer Range	PCR Detection of *Y. pestis* DNA
West Kazakhstan	127	7	85.2	1:20–1:160	Negative
Atyrau	325	14	70.6	1:20–1:320	Negative
Mangystau	685	21	75.4	1:20–1:320	Negative
Aktobe	250	10	91.2	1:20–1:320	Negative
Kyzylorda	368	23	71.2	1:20–1:320	Negative
Turkestan	363	14	70.1	1:20–1:320	Negative
Zhambyl	325	11	67.2	1:20–1:160	Negative
Almaty	283	15	74.3	1:20–1:320	Negative

Abbreviations: PCR, polymerase chain reaction; *Yersinia pestis*.

**Table 4:** Camel population, vaccination coverage, and epizootological control measures in Kazakhstan (2020–2025).

Year	Camel Population (Thousand Heads)	Vaccinated Animals (*n*)	Vaccination Coverage (%)	Plague Epizootic Area (Thousand km^2^)	Protective Zones (*n*)	Protective Zones Area (km^2^)
2020	227.7	87,875	38.6	7.1	152	344.4
2021	243.4	91,547	37.6	5.4	116	354.1
2022	253.7	89,546	35.3	3.0	196	595.0
2023	264.9	90,268	34.1	3.8	154	546.4
2024	280.5	89,864	32.0	5.7	177	394.0
2025	304.0	155,059	51.0	8.9	180	423.2

## Data Availability

The data presented in this study are available within the article. Additional data supporting the findings of this study are available from the corresponding author upon reasonable request.
